# Metal Ion Release, Clinical and Radiological Outcomes in Large Diameter Metal-on-Metal Total Hip Arthroplasty at Long-Term Follow-Up

**DOI:** 10.3390/diagnostics10110941

**Published:** 2020-11-12

**Authors:** Assunta Pozzuoli, Antonio Berizzi, Alberto Crimì, Elisa Belluzzi, Anna Chiara Frigo, Giorgio De Conti, Annamaria Nicolli, Andrea Trevisan, Carlo Biz, Pietro Ruggieri

**Affiliations:** 1 Laboratory of Musculoskeletal Pathology and Oncology, Orthopaedic and Traumatologic Clinic, Department of Surgery, Oncology and Gastroenterology, University of Padova, Via Giustiniani 3, 35128 Padova, Italy; 2 Orthopaedic and Traumatologic Clinic, Department of Surgery, Oncology and Gastroenterology, University of Padova, Via Giustiniani 3, 35128 Padova, Italy; antonio.berizzi@unipd.it (A.B.); albe.crim@gmail.com (A.C.); carlo.biz@unipd.it (C.B.); pietro.ruggieri@unipd.it (P.R.); 3 Epidemiology and Public Health Unit, Department of Cardiac Thoracic Vascular Sciences and Public Health, Biostatistics, University of Padova, Via Loredan 18, 35131 Padova, Italy; annachiara.frigo@unipd.it; 4 Department of Radiology, University Hospital of Padova, Via Giustiniani 2, 35128 Padova, Italy; giorgio.deconti@aopd.veneto.it; 5 Laboratory of Industrial Toxicology, Department of Cardiac Thoracic Vascular Sciences and Public Health, University of Padova, Via Giustiniani 2, 35128 Padova, Italy; annamaria.nicolli@unipd.it (A.N.); andrea.trevisan@unipd.it (A.T.)

**Keywords:** total hip arthroplasty, cobalt, chromium, metal-on-metal, ceramic-on-ceramic

## Abstract

Total hip arthroplasty (THA) with metal-on-metal (MoM) bearings have shown problems of biocompatibility linked to metal ion release at the local level causing an adverse reaction to metal debris (ARMD) and at a systemic level. The aim of this study was to evaluate clinical and radiological outcomes, and metal ion concentrations in the blood and urine of patients who underwent THA with the LIMA Met-Met hip system. Patients with ceramic-on-ceramic (CoC) bearings were included as a control group. In this study, 68 patients were enrolled: 34 with MoM THAs and 34 with CoC THAs. Patients were evaluated clinically (Harris Hip Score, SF-36) and radiologically at a median of 7.4 years after surgery. Whole blood and urinary cobalt and chromium levels were also assessed. Both types of implants were comparable in terms of clinical and functional results. Ion levels were significantly higher in the MoM group compared with CoC group 7 years after surgery. No correlations were found between metal ion levels and patient demographics, functional and radiological outcomes, and prosthesis features. Patient monitoring is thus advised to establish if prosthesis revision is necessary, especially in the case of MoM THA.

## 1. Introduction

**Figure 1:**
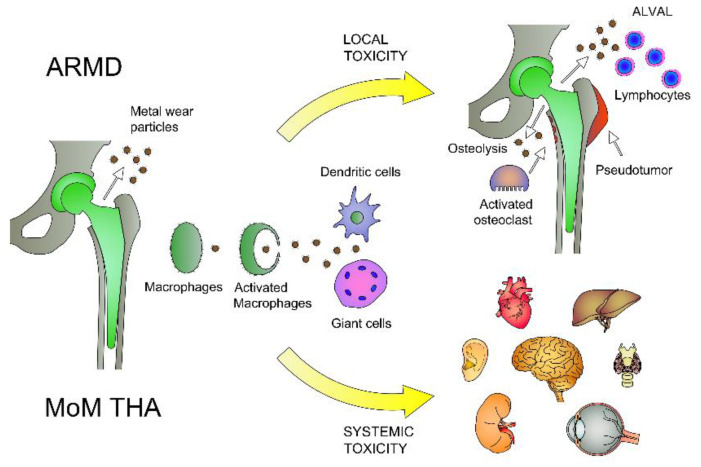
Local and systemic toxicity of metal-on-metal (MoM) metal debris. Metal debris, released from the implant by corrosion or wear, causes local periprosthetic alterations called adverse reaction to metal debris (ARMD), mediated by different types of cells including macrophages, osteoclasts, giant cells, dendritic cells and lymphocytes. Tissue alterations can cause osteolysis (induced by osteoclasts and macrophages) at the bone-level, or pseudotumor formation and aseptic lymphocyte–dominated vasculitis-associated lesions (ALVAL) at the soft tissue-level. Periarticular metal debris can be disseminated to different organs through circulatory and lymphatic systems causing systemic toxicity.

Total Hip arthroplasty (THA) represents one of the most successful surgical operations in terms of cost-effectiveness and improvement of quality of life [1]. In 2017, Germany (309 per 100,000 population), and Switzerland (307 per 100,000 population), were among the countries with the highest rates for THA [2]. The demand of THA is expected to increase, reaching 572,000 by the year 2030 in the United States [3]. Three main types of implant systems have been developed with the purpose of obtaining better clinical outcomes with a lower complication rate and a longer prosthesis survival: metal-on-polyethylene (MoP), metal-on-metal (MoM) and ceramic-on-ceramic (CoC) [4]. MoP implants provide a safe, predictable and cost-effective hip bearing surface for most patients; however, this type of prosthesis releases polyethylene (PE) debris, causing the production of cytokines and proteolytic enzymes [5]. PE debris, the main cause of THA failure, triggers periprosthetic osteolysis with aseptic loosening as endpoint [5]. In the late 1990s, a new generation of MoM systems became the primary choice for THA because of a greater diameter of the femoral heads with a lower dislocation rate, improved hip stability, greater range of motion (ROM) and better performance than MoPs [6,7]. It has been estimated that more than 1000,000 THAs with MoM bearings have been implanted worldwide since 1996 [8]. However, the main drawback of MoM bearings is the production of metal debris due to the combined effect of mechanical and corrosive wear [9]. MoM implants can release metal particles, in particular cobalt (Co) and chromium (Cr) [10], causing the phenomenon called adverse reaction to metal debris (ARMD) at the local level (Figure 1) [11,12]. Metal ions can also be released into the blood causing systemic toxicity (Figure 1) [12].

To address wear and friction problems, new generations of CoC bearing systems have been developed. The use of CoC bearings has several advantages: improved strength and toughness, scratch resistance, superior lubricating properties and biologically inert debris compared to the toxic metals released from MoMs and toxic PE debris from MoPs [4]. Even though metal release from MoM implants is well known, long-term clinical follow-up and studies are still ongoing.

Evaluation of systemic concentrations of metal ions has been proposed to detect wear, malposition of prosthesis components, poor hip function, early failure [13], local toxicity and systemic toxicity; it is also useful for follow-up [14]. However, there is still no international consensus on the surveillance protocols to follow-up MoM THA [4,15]. In addition, no clear thresholds of ion levels are available to identify poorly performing hips and patients at greater risk of developing ARMD [16,17,18,19].

Because of the safety concerns, and according to national and international societies’ and regulatory authorities’ guidelines [18,20,21,22], the present study with a long-term follow-up was conducted to monitor patients who underwent surgery for THA with the LIMA Met-Met hip system at our institution. The aim of the study was to evaluate clinical and radiological outcomes and cobalt and chromium metal ion concentrations in the blood and urine of patients who underwent MoM THA, comparing the data with those of CoC THA as controls. The hypothesis was that the metal ion levels of MoM THA were higher compared to CoC THA but clinical and radiological outcomes were comparable between the two groups.

### 1.1. Study Population

From January 2007 to July 2011, a series of consecutive patients who underwent THA were recruited. Patients with CoC hip implants who underwent surgery in the same period were included in the study as a control group. Inclusion criteria were patients with MoM or CoC THA and written informed consent. Exclusion criteria were the inability to give written informed consent, refusal to participate in the study, bilateral implant, severe kidney disease, immunological diseases and serious disability limiting walking.

**Figure 2:**
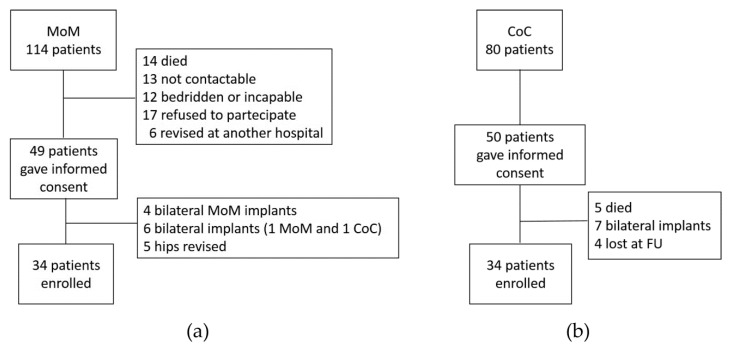
Flowchart summarizing patients involved in the study: (**a**) MoM, metal-on-metal bearing; (**b**) CoC, ceramic-on-ceramic bearing. FU = follow-up.

This study has been approved by the local Ethics Committee (CESC Code: 2561 P; 12th March 2012) and was carried out in accordance the tenets of the Helsinki Declaration, and all patients provided informed consent prior to be included into the study. Two hundred and nine hip replacement operations were performed in 194 patients at our institution. One hundred and twenty-five MoM THAs were implanted in 114 patients and 84 CoC in 80 patients. In 2012, a case-control study was begun that included 34 patients of the original 114 patients (125 hips) provided with the LIMA Met-Met System for THA and 34 of the original 80 patients (84 hips) with the LIMA ceramic-ceramic system, subsequently indicated as the MoM and CoC groups, respectively (Figure 2).

Five patients had a revision of the MoM implant: two patients due to ARMD [23] and three for mechanical complications.

The demographic and clinical features of the patients are reported in Table 1, which shows that the characteristics of the two groups were comparable.

The MoM patients were 11 males and 23 females (mean age 63.8 ± 14.6 years and 67.2 ± 7.9 years, respectively), while the CoC patients were 13 males and 21 females (mean age 69.0 ± 6.8 years and 68.3 ± 8.2 years, respectively). Fracture was the main cause of THA in both groups, followed by hip osteoarthritis. Between surgery and enrolment, the median interval was 7.4 (range 3.2–10.0) years for the MoM group and 7.4 (range 3.8–9.3) years for the CoC group (*p* = 0.61). The median diameter of the femoral head was 46 (range 42–58) mm in the MoM group and 36 (range 32–40) mm in the CoC patients (*p* < 0.0001). It was significantly larger in men (median 50, range 42–58) compared to women (median 46, range 42–54; *p* = 0.001) in the MoM group, but not in the CoC group (men median 40, range 36–40; women median 36, range 32–40; *p* = 0.06).

### 1.2. Hip Implants

Two types of total hip prostheses were implanted: the LIMA Met-Met System and the LIMA Ceramic-Ceramic System (LIMA Corporate, Villanova di San Daniele del Friuli (UD), Italy). The LIMA Met-Met System is a forged implant of high-carbon cobalt-chromium with a low-profile acetabular floor and a reduced thickness of the cup. The low profile allows the use of large diameter heads that reduce the risk of dislocation, have low wear and increase the range of movement (ROM). The Met-Met acetabular cup is made of an alloy of Co, Cr and molybdenum (Mo), porous titanium (Ti) and hydroxyapatite (HA). The head is made of CoCrMo. A head-neck taper 12/14 is present, composed by Ti_6_Al_4_V.

The LIMA Ceramic-Ceramic System has two different types of cups (Delta-TT or Delta-PF) that are implanted. Both cups have the same geometry composed of a titanium alloy of titanium, aluminium and vanadium (Ti_6_Al_4_V), porous Ti and HA, and have a hemispherical design for use without cement and an oversized diameter for the appropriate press-fit [24]. The ceramic heads are the BIOLOX *forte* or BIOLOX *delta* (CeramTec, Plochingen, Germany). A ceramic insert BIOLOX *delta* is placed between the acetabular cup and the head.

All patients received a femoral stem manufactured by LIMA in three forms: PLS, C2 and Modulus stems of Ti_6_Al_4_V.

### 1.3. Surveillance Follow-Up Program

Patients meeting the inclusion criteria were recruited in the surveillance program after having signed the informed consent form. All patients were followed up prospectively at 6, 12 and 24 months after recruitment. In this study, data from the longest follow-up, at 24 months from recruitment, are presented. At each visit, clinical, radiological and laboratory evaluations were performed.

### 1.4. Clinical Evaluation

Complete clinical histories including demographic data (gender, age at surgery, body mass index (BMI), diagnosis, physical examination and passive and active ROM) were recorded. Furthermore, diet, smoking habits, history of allergy, use of drugs, occupational exposure to Co and Cr and physical activity were assessed. Both groups of patients were clinically evaluated using the Harris Hip Score (HHS), and the Short Form 36 (SF 36) and its component summary scales concerning physical health (PCS) and mental condition (MCS) [25,26]. Pain was recorded as a component of the HHS. In the HHS, pain subscale is divided in six points: None, or ignores it (grade 0); Slight, occasional, no compromise in activity (grade 1); mild pain, no effect on average activities, rarely moderate pain with unusual activity, may take aspirin (grade 2); Moderate pain, tolerable but makes concessions to pain. Some limitations of ordinary activity or work. May require occasional pain medication stronger than aspirin (grade 3); Marked pain, serious limitation of activities (grade 4) and Totally disabled, crippled, pain in bed, bedridden (grade 5).

### 1.5. Radiological Evaluation

**Figure 3:**
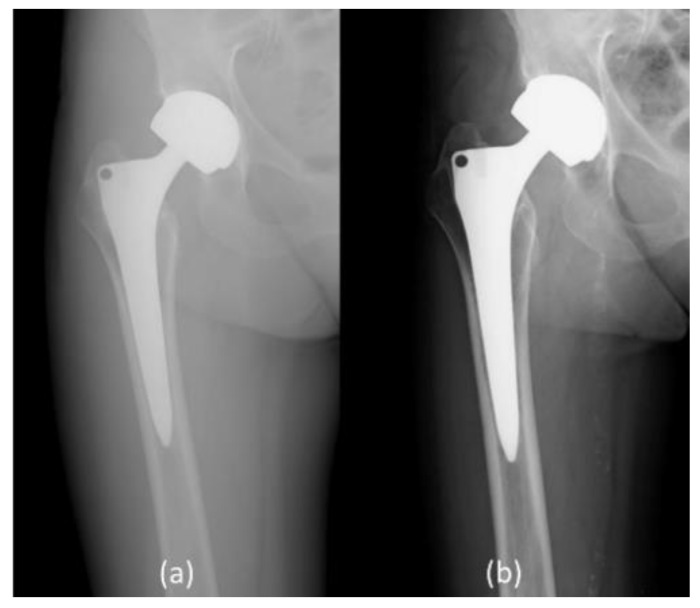
F.A., 81-year-old female, Metal-on-Metal Total Hip arthroplasty for primary arthrosis, right hip X-ray. (**a**) Post-operative follow-up, one month (**b**) Post-operative follow-up, 7 years (24 months after recruitment).

**Figure 4:**
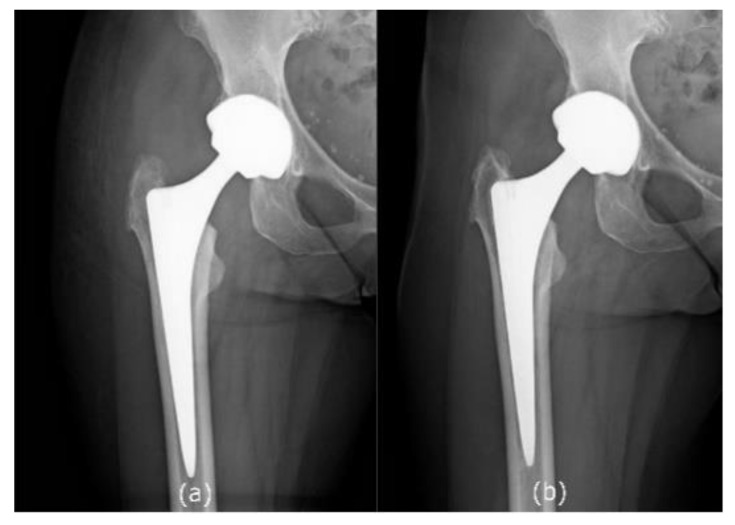
V.D., female, 58 - year-old, Ceramic-on-Ceramic Total Hip arthroplasty for femoral neck fracture, right hip X-ray: (**a**) Post-operative follow-up, one month (**b**) Post-operative follow-up, 5 years (24 months after recruitment).

Anteroposterior and lateral radiographs of the operated hip were performed at the follow-up visit to evaluate implant loosening. Two representative images of postoperative x-ray at one month after surgery and at the last follow-up (24 months after recruitment) are presented in Figure 3 and Figure 4.

The presence of osteolysis was evaluated using Gruen and DeLee classifications [27,28]. Additionally, the inclination angle of the acetabular component was measured according to Sharp [29]; an angle higher than 55° was considered steep [30]. Femoral offset (FO) was measured as the distance from the centre of rotation of the femoral head to a line dissecting the long axis of the femur and the difference between FO of the operated hip and contralateral hip (DFO) was calculated. Normal FO was between −5 and +5 mm, while decrease FO and increased FO were ≤−5 mm and ≥+5 mm compared to the contralateral hip, respectively [31].

All measurements were performed using the software for the management of radiological images Medstation (Exprivia, Italy) on a diagnostic LCD CORONIS 5 MP display monitor (Barco, Rome, Italy).

### 1.6. Metal Ion Measurement

A venous whole blood sample and one spot specimen of urine were obtained at the follow-up visit. All samples were frozen and stored at −20 °C until analysis. All of the analyses were performed by a certified industrial toxicological laboratory (nr IT-24863). All the measurements were performed in duplicate.

Metal ion levels were assessed by a graphite furnace atomic absorption spectrophotometer (Perkin-Elmer AAnalist 600) with Zeeman-effect for background correction. The detection limits of metals were whole blood Co (CoB) and urinary Co (CoU) 0.56 µg/L, whole blood Cr (CrB) 0.1 µg/L and urinary Cr (CrU) 0.08 µg/L. According to the MHRA, the cut-off for metal ion levels for risks of clinical complications related to metal ion release is 7 µg/L [20]. In case of levels above this threshold (three patients), metal ion measurement was repeated after 3 months.

All of the urinary values were normalised with urinary creatinine to eliminate the influences of concentration/dilution due to possible confounders such as renal physiology and metal clearance, hydration state and perspiration [14]. Urinary creatinine was determined using the basic-picrate Jaffe reaction [14]. All of the values were expressed as μg/g of creatinine [14].

### 1.7. Statistical Analysis

Quantitative variables were summarized with mean, standard deviation, median and range, while qualitative variables with count and percentage of patients in each category. Normality of quantitative variables was visualized by a box-plot and evaluated by the Shapiro–Wilk test. Comparison of not normal quantitative variables was performed by the Mann-Whitney U or Kruskal–Wallis test.

Possible correlations between metal ions released in patients with MoM and age at surgery, BMI, BSA, prosthetic head diameter, acetabular angle inclination and clinical scores were evaluated with Spearman’s Correlation Coefficient.

Separately for the two groups of patients, association of metal ions with gender, use of dietary supplementation and radiographic alterations was evaluated with the Mann–Whitney U Test.

Comparison between metal ions and smokers, non-smokers and ex-smokers was done separately for the three groups with the Kruskall–Wallis test. Association between stem type and radiological alterations was done with the Fisher’s exact test.

Statistical analyses were performed with SAS 9.4 (SAS Institute Inc., Cary, NC, USA) for Windows. A *p*-value lower than 0.05 was considered indicative of statistical significance.

## 2. Results

### 2.1. Clinical Outcomes

All of the clinical outcomes (HHS, pain score and SF-36) were not significantly different between the two groups (Table 2).

Regarding HHS, most of the patients showed good or excellent values in both groups. The median HHS was 89.9 (50.9–97.0) points in the MoM group and 88.0 (48.9–97.0) points in the CoC group (Table 2). No difference was found comparing the two groups (*p* = 0.70). The pain score was not significantly different between the two groups. Almost half of the patients were asymptomatic, while about thirty percent experienced slight pain both in MoM and CoC groups. About 20% of MoM patients had mild to moderate pain compared to about 10% of CoC patients. Only one patient in the CoC group felt marked pain, but no patients experienced disabling pain (Table 2). None of the patients had grade 5 of pain. Comparing MoM to CoC groups, the SF36 summary scales, PCS and MCS were not significantly different (*p* = 0.20 and *p* = 0.94, respectively).

### 2.2. Radiological Outcomes

Periprosthetic osteolysis was present in two-thirds of the MoM group (67.6%) upon radiological examination, while only in about one-fifth of the CoC group (17.6%). This difference was statistically significant (*p* < 0.0001) (Table 3).

The average inclination angle was comparable between MoM and CoC groups (median 40.0 degrees and 40.5 degrees, respectively) (Table 3). No acetabular components were implanted with more than 55° of inclination in either group. No differences were observed regarding the postoperative FO between the two groups. Moreover, the DFO between the operated and the contralateral hip was comparable between the two groups. In MoM group only four patients (11.8%) exceeded the normal FO showing an increased FO ≥ +5 mm. Only one patient (2.9%) in the CoC group had a decreased FO ≤ −5 mm. No correlations were found between FO, DFO and metal ion release.

### 2.3. Metal Ion Release in Blood and Urine Samples

**Figure 5:**
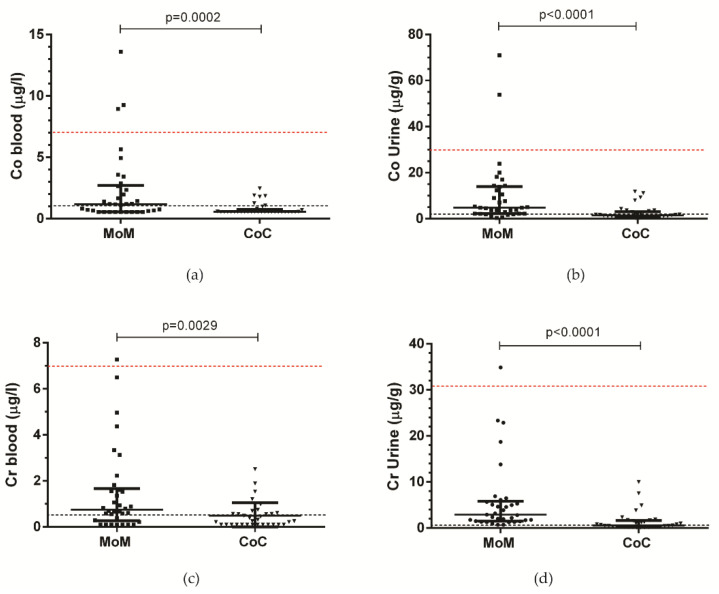
Scatter dot plot (median with interquartile range) of metal ion levels in MoM and CoC groups. Cobalt ion level in whole blood (**a**) and urine (**b**). Chromium ion level in whole blood (**c**) and urine (**d**). Urinary values are expressed as μg/g of creatinine. Dotted lines represent the laboratory upper limits of the normal range in the general population for cobalt and chromium in whole blood (1 and 0.5 µg/L, respectively). Dotted red lines represent the safety threshold of 7 µg/L for Co and Cr in whole blood. In urinary levels normalized to creatinine (**b** and **d**), dotted lines represent the normal values of CoU < 2 µg/g and CrU < 0.05 according to the National Institute for Research and Safety (Institut national de recherche et sécurité (INRS)). Dotted red lines represent the safety threshold of 30 µg/L for Co and 31 for Cr in urine.

Whole blood metal ion levels were significantly higher in the MoM group compared to the CoC group, as shown in Table 4 and Figure 5.

The levels of Co and Cr were compared to the guidelines of the Medicines and Healthcare products Regulatory Agency (MHRA), which states that the cut-off for metal ion levels for risks of clinical complications related to metal ion release is 7 µg/L. Cobalt level in whole blood (CoB) was higher than 7 µg/L in three patients, while the chromium level in whole blood (CrB) was above the cut-off only in one patient in the MoM group; no CoC patients had metal ion levels that exceeded this amount. No patients had blood levels higher than those associated with metallosis, which is 19 µg/L for CoB and 17 µg/L for CrB [14]. CoB had a positive correlation with CrB only in the MoM group (r = 0.591, *p* = 0.0002) but not in CoC patients. In addition, urinary metal ion levels were significantly higher in the MoM group compared to the CoC group (Table 4).

A threshold in urine of 30 µg for Co and 21 µg for Cr, adjusted to creatinine, based on the threshold in whole blood of 7 µg/L for these two metals has been proposed [14]. Cobalt level in urine (CoU) exceeded that threshold only in two females and chromium level in urine (CrU) in three females of the MoM group, and never in CoC patients. CoB correlated with CoU (r = 0.690, *p* < 0.0001) and CrU (r = 0.659, *p* < 0.0001) in the MoM group. Similarly, CrB correlated with CoU (r = 0.587, *p* = 0.0003) and CrU (r = 0.729, *p* < 0.0001). In the CoC group, a correlation was found only between CoU and CrU (r = 0.699, *p* < 0.0001).

### 2.4. Comparison Between Asymptomatic and Symptomatic Patients

A comparison between asymptomatic and symptomatic patients was carried out in each group and between the two groups.

A statistically significant difference was found comparing metal release in blood and urine of asymptomatic MoM vs. asymptomatic CoC and symptomatic MoM vs. symptomatic CoC, except for CrB in symptomatic MoM vs symptomatic CoC. No differences were observed comparing the metal levels of asymptomatic vs. symptomatic patients of the same prosthesis type (Table 5).

Regarding HHS, a difference was found when asymptomatic and symptomatic patients of the same prosthesis type were compared. Regarding SF36 score, differences were reported comparing the PCS scale of asymptomatic CoC vs. symptomatic CoC, the MCS scale of asymptomatic MoM vs. asymptomatic CoC and asymptomatic MoM vs. symptomatic MoM (Table 6).

### 2.5. Influence of Patient Demographic and Clinical Features on Metal Ion Release

Significant differences were found in urinary metal ion levels according to gender: ion release was higher in females versus males. Specifically, a difference was found for CoU (*p* = 0.011) and CrU (*p* = 0.049) in the MoM group. In the CoC group, there was a difference for CoU (*p* = 0.037). Smoking significantly affected ion concentrations for CoU (*p* = 0.016) and CrU (*p* = 0.002) in MoM patients but not in CoC patients. No significant differences were found comparing metal ion release and diet supplementation, BMI, drug use, physical activity, and x-ray features and stem in both groups.

Between BMI and CoU in the MoM group, there was a negative correlation (r = −0.420, *p* = 0.013). No correlations were found between metal ion levels and clinical and radiological outcomes. Regarding prosthesis features, a negative correlation was found only between head diameter and CoU in the CoC group (r = −0.348, *p* = 0.043).

## 3. Discussion

The use of MoM bearings has been discontinued due to biological concerns, local and systemic release of metal ions [32,33,34,35] with frequent reports of ARMD [36]. The diagnosis of ARMD in MoM THA is a multifactorial process that involves mechanical, radiological and clinical factors [37]. Metal ion release could be affected by several aspects including differences in manufacturing and metallurgy as well as metal corrosion [38]. Therefore, it is important to study every type of prosthesis, as stated by other authors who highlighted the importance of implant design features [39], recommending implant-specific thresholds for metal ions in monitoring patients with MoM implants [40,41]. Moreover, the peak of metal release in whole blood has been reported to be very different, ranging from around 9–12 months to 4–5 years after surgery, followed by a plateau level thereafter [42].

The present study was conducted to assess the clinical and functional performance of the LIMA Met-Met Hip System through the evaluation of Co and Cr release and its correlations with patient demographics, functional and radiological outcomes, and prosthesis features in comparison with a control group, CoC THA patients, at long-term follow-up.

Baseline characteristics (age, gender and BMI) were not significantly different between MoM and CoC groups, confirming that the two groups were comparable. Both MoM and CoC groups obtained good to excellent clinical results, as shown by HHS and SF36 scores, confirming the good mechanical and functional features of MoM implants compared to CoC ones, in agreement with several published studies and with our hypothesis [43,44,45,46]. However, some authors reported worse clinical outcomes of MoM compared with CoC, demonstrating the higher revision rate of MoM implants [47,48]. According to Hussey et al., [49] who developed a clinical scoring system that uses HHS for stratifying MoM patients into low (80–100), moderate (70–79) and high risk (<70) of implant revision, most of our patients were at low risk of implant failure, showing HHS greater than 80 points.

No differences were found considering pain. Almost half of the patients were asymptomatic, while 30% experienced slight pain in both groups.

Comparing patients based on pain, HHS was significantly different in asymptomatic and symptomatic patients both in MoM and CoC groups. This is not surprising because pain was obtained by the pain subscale of HHS. Regarding SF36, pain had an impact on physical perception only in the CoC group; in the MoM group, it had an impact at a mental level, but the physical component was not affected.

Regarding the radiological evaluation, the MoM group showed a higher presence of osteolysis compared to the CoC group, as reported by Higuchi et al. [50]. Nevertheless, MoM patients also have good functional outcomes [51,52]. Postoperative FO was in the normal range. Most of the patients had a DFO in the normal range except for four MoM patients and one CoC patient. No statistically significant correlations were found between DFO and metal ion release. Despite the fact that these parameters could have a role in material loss [51,52], no correlations were highlighted between FO and DFO and the metal ion release in our study. These data are in accordance with a recent study published by Pomorey et al. demonstrating no relationship between FO and wear by measuring circulating metal ion levels in MoM THA [31].

In agreement with previous literature [9,33,44,53,54,55], we found that Co and Cr levels, assessed both in whole blood and urine, were significantly higher in the MoM group compared with the CoC group, in line with our hypothesis. Some authors have reported that metal ions released from large diameter MoM THAs could originate not only at the articulating surfaces but also at the metallic trunnion surfaces, the head-neck taper or adapter [11,16].

Considering the CoC group, most of the patients had whole blood metal ion release within the normal range set by our reference laboratory.

All urinary metal levels were significantly higher in the MoM group than in CoC patients.

We found increased urinary metal ions in females versus males in both groups. Female gender, along with small femoral head size, is considered a risk factor for wear, metal ion release and ARMD [19], but the recent literature is conflicting. Some authors have reported increased blood metal ion levels in MoM THAs for females [19,56], while others did not find an effect of gender on metal ion release [57,58]. However, although women had femoral head diameters significantly smaller than men in the MoM group, no association was found with metal ion release [33,59]. Nevertheless, females have different hip anatomy and biomechanics with increased acetabular component inclination, reduced joint size and increased range of motion [60]. They also have a different metal ion metabolism in terms of excretion and cellular and extracellular storage [61,62].

In the present study, all implanted femoral heads were large diameter, 36 mm or greater, in all patients of the MoM group. No differences were found between metal ion levels and gender and femoral head size in CoC patients. The association between implant head size and metal ion release is not clear in the literature, with studies reporting higher release from large diameter head size THAs [33,59], no association with head size [32,63] or higher release from small head size THAs [56,58].

Furthermore, blood and urinary metal ion levels were not affected by patient age, physical activity, acetabular inclination angle, x-ray features (even though MoM patients showed a higher percentage of osteolysis than the CoC group) and types of stem in both groups according to other studies [30,64,65].

Our surveillance protocol foresaw that patients were usually monitored annually unless the appearance of new symptoms or in presence of metal ion levels exceeding the threshold. In this case, the patient underwent a clinical, radiological imaging of the 2nd level and laboratory evaluation every 3 months. In addition, thyroid, liver and heart functionality were performed to exclude organ damage due to the systemic dissemination of metal debris. Only three patients of this cohort exceeded the metal cut-off and thus, were evaluated every 3 months. However, these patients had good clinical outcomes and did not present altered organ functionality.

Finally, considering that patients are now evaluated at longer intervals from primary surgery, the long-term systemic effects of metal ion release should be considered, particularly the potential oncological consequences. In our study, only two patients had a history of solid cancer (not sarcoma in the periprosthetic tissue). These data are in agreement with the literature [66,67].

There were some limitations in this study. Even though it was conducted prospectively, no preoperative data are available. This was because the study started after awareness of the safety concerns related to higher revision rates and adverse reactions of some MoM hip implants. Moreover, the present work involved a small sample size, even if the number is in line with the majority of published studies [44,48,53,68,69], limiting the possibility to highlight more correlations between metal ion levels and clinical and radiological scores evaluated. The impact of titanium release cannot be excluded as we did not measure its levels. It should be recognized that the recent research on titanium release points out a controversy regarding the use of certain titanium alloys in hip arthroplasty [70]. However, it should be noted that MHRA and FDA agencies still do not advise to monitor this metal [20,21,71].

Nonetheless, the present study has important strengths. To the best of our knowledge, this is the first report of Co and Cr release in whole blood from large-diameter LIMA Met-Met Hip System arthroplasties. Moreover, we enrolled CoC THA as a control group. Metal ion levels were measured in whole blood, which is considered more accurate than serum [72] and recommended by the MHRA [20]. The use of whole blood reduces laboratory manipulation and consequent risk of contamination, and better represents systemic exposure [72]. Additionally, all urinary metal ions levels were adjusted to creatinine to eliminate the dilution effect of spot urine samples [14]. All of the toxicological analyses were performed by an accredited toxicological laboratory because laboratories often use different techniques making comparison of metal levels of limited clinical relevance [16,73].

## 4. Conclusions

In conclusion, to the best our knowledge, this is the first report on Co and Cr levels in whole blood released from large-diameter LIMA Met-Met Hip System prostheses, showing that MoM implants have a higher metal release than CoC group at long-term follow-up.

Therefore, evaluation of metal ion release in MoM THA patients is mandatory for both patient safety and implant survival, and interdisciplinary cooperation is necessary between orthopaedic surgeons and other specialties, mainly radiology and laboratory medicine.

We will continue to follow this cohort of patients, updating the long-term results and advising patients who quit the surveillance protocol to undergo metal ion and radiological evaluations every year, especially if there are clinical changes.

## Figures and Tables

**Table 1:** Patients demographic and clinical characteristics.

Characteristics	MoM (*n* = 34)	CoC (*n* = 34)	*p*-Value
Women *n* (%)	23 (67.6)	21 (61.8)	0.80 *
Men *n* (%)	11 (32.4)	13 (38.2)	
Age (years) Mean (SD)	66.1 (10.4)	68.6 (7.6)	0.26 ¶
Left side *n* (%)	19 (55.9)	18 (52.9)	1.00 *
Femoral head (mm) Median (Range)	46.0 (42.0–58.0)	36.0 (32.0–40.0)	<0.0001 °
BMI (Kg/m²) Median (Range)	24.3 (18.9–38.2)	25.5 (19.5–38.9)	0.14 °
Dietary supplements *n* (%)	6 (17.6)	4 (11.8)	0.73 *
Smoke *n* (%)	10 (29.4)	13 (38.2)	0.61 *
Allergies *n* (%)	11 (32.4)	5 (14.7)	0.15 *
Drugs *n* (%)	29 (85.3)	32 (94.1)	0.43 *
Job exposure *n* (%)	4 (11.8)	2 (5.9)	0.67 *
Physical activity *n* (%)	18 (54.9)	13 (38.2)	0.33 *
Diagnosis *n* (%)			0.12 *
Bone fracture	17 (50.0)	22 (64.7)	
Osteoarthritis	13 (38.2)	12 (35.3)	
Necrosis	4 (11.8)	0 (0.0)	
Follow-up time after surgery (years) Median (Range)	7.4 (3.2–10.0)	7.4 (3.8–9.3)	0.61 °

SD standard deviation, BMI body mass index. * Fisher’s exact test. ¶ Student’s t test. ° Mann-Whitney U test.

**Table 2:** Patient clinical outcomes.

Outcomes	MoM (*n* = 34)	CoC (*n* = 34)	*p*-Value
Harris Hip score Median (Range)	89.9 (50.9–97.0)	88.0 (48.9–97.0)	0.70 °
Pain *n* (%)			0.24 *
Grade 0	14 (41.2)	19 (55.9)	
Grade 1	12 (35.3)	11 (32.4)	
Grade 2	6 (17.6)	1 (2.9)	
Grade 3	2 (5.9)	2 (5.9)	
Grade 4	0 (0.0)	1 (2.9)	
Grade 5 SF-36 Median (Range)	0 (0.0)	0 (0.0)	
PCS Scale	41.0 (20.7–58.4)	47.7 (23.9–60.4)	0.20 °
MCS Scale	53.9 (12.0–64.1)	51.7 (23.3–66.1)	0.94 °

SF 36 = 36-item Health Survey; PCS = Physical Component Score; MCS = Mental Component Score. Pain, recorded as a component of the HHS, is expressed as number of patients (*n*) and percentage in brackets. * Fisher’s exact test. ° Mann–Whitney U test.

**Table 3:** Radiological outcomes.

Outcomes	MoM (*n* = 34)	CoC (*n* = 34)	*p*-Value
Inclination angle (°) Median (Range)	40.0 (9.0–55.0)	40.5 (12.0–55.0)	0.93 °
Periprosthetic osteolysis *n* (%)	23 (67.6)	6 (17.6)	<0.0001 *
Postoperative Femoral offset (mm) Median (Range) DFO	41.9 (32.5–55.3) 1.6 (−4.9–8)	41.8 (33.6–54.7) 1.1 (−6–5)	0.565 ° 0.749 °

DFO = femoral offset difference compared to contralateral hip. * Fisher’s exact test. ° Mann–Whitney U test.

**Table 4:** Whole blood and urine cobalt and chromium levels.

Metals	MoM (*n* = 34)	CoC (*n* = 34)	*p*-Value
Cobalt B (µg/L)	1.2 (0.6–13.6)	0.6 (0.6–2.5)	0.0002 *
Cobalt U/Creatinine (µg/g)	4.8 (0.4–71.0)	1.4 (0.2–11.8)	<0.0001 *
Chromium B (µg/L)	0.8 (0.1–7.3)	0.3 (0.1–2.5)	0.0029 *
Chromium U/Creatinine (µg/g)	2.9 (0.7–34.9)	0.6 (0.1–10.0)	<0.0001 *

U = urine, B = blood, SD = standard deviation, data were reported as median (range). * Mann–Whitney U test.

**Table 5:** Metal ion concentration comparing asymptomatic and symptomatic patients.

Metals	MoM		CoC		*p*–Value
	Asymptomatic (*n* = 14)	Symptomatic (*n* = 20)	Asymptomatic (*n* = 19)	Symptomatic (*n* = 15)	
Cobalt B (µg/L)	0.7 (0.56–9.25)	1.2 (0.6–13.6)	0.6 (0.56–2.5)	0.6 (0.6–1.9)	a 0.030 b 0.003 c 0.458 d 0.950
Cobalt U/Creatinine (µg/g)	4.4 (0.85–23.9)	4.9 (0.4–71.0)	1.2 (0.3–9.3)	1.8 (0.2–11.8)	a 0.001 b 0.012 c 0.649 d 0.107
Chromium B (µg/L)	0.9 (0.1–6.5)	0.6 (0.1–7.3)	0.3 (0.1–1.5)	0.3 (0.1–2.5)	a <0.0001 b 0.261 c 0.292 d 0.617
Chromium U/Creatinine (µg/g) Median (Range)	2.9 (1.24–23.4)	3.1 (0.7–34.9)	0.3 (0.1–10.0)	1.2 (0.1–7.5)	a <0.0001 b 0.003 c 0.834 d 0.054

Data are reported ad median (range). Table. *Cont.* a asymptomatic MoM vs. asymptomatic CoC. b symptomatic MoM vs. symptomatic CoC. c asymptomatic MoM vs. symptomatic MoM. d asymptomatic CoC vs. symptomatic CoC. All statistical analyses were performed using Mann–Whitney U test.

**Table 6:** Clinical and radiological outcomes comparing asymptomatic and symptomatic patients.

Outcomes	MoM		CoC		*p*–Value
	Asymptomatic (*n* = 14)	Symptomatic (*n* = 20)	Asymptomatic (*n* = 19)	Symptomatic (*n* = 15)	
HHS	94.4 (86.8–97.0)	81.5 (50.9–92.9)	92.1 (64.3–97.0)	81.8 (48.8–94.9)	a 0.412 b 0.641 c 0.000029 d 0.006
SF-36					
PCS Scale	47.9 (29.7–57.7)	38.7 (20.7–58.4)	52.7 (23.8–60.4)	39.5 (27.7–59.1)	a 0.274 b 0.841 c 0.107 d 0.028
MCS Scale	55.4 (49.8–55.4)	42.4 (12.0–64.1)	52.6 (31.3–59.8)	50.1 (23.3–66.1)	a 0.032 b 0.182 c 0.013 d 0.959

HHS = Harris Hip Score; PCS = Physical Component Score; MCS = Mental Component Score. Data are reported as median (range). a asymptomatic MoM vs. asymptomatic CoC b symptomatic MoM vs. symptomatic CoC c asymptomatic MoM vs. symptomatic MoM d asymptomatic CoC vs. symptomatic CoC. All statistical analyses were performed using Mann–Whitney U test.
